# Pioneer cells established by the [*SWI*^+^] prion can promote dispersal and out-crossing in yeast

**DOI:** 10.1371/journal.pbio.2003476

**Published:** 2017-11-14

**Authors:** Gregory A. Newby, Susan Lindquist

**Affiliations:** 1 Department of Biology, Massachusetts Institute of Technology, Cambridge, Massachusetts, United States of America; 2 Whitehead Institute for Biomedical Research, Cambridge, Massachusetts, United States of America; 3 Howard Hughes Medical Institute, Massachusetts Institute of Technology, Cambridge, Massachusetts, United States of America; New York University, United States of America

## Abstract

To thrive in an ever-changing environment, microbes must widely distribute their progeny to colonize new territory. Simultaneously, they must evolve and adapt to the stresses of unpredictable surroundings. In both of these regards, diversity is key—if an entire population moved together or responded to the environment in the same way, it could easily go extinct. Here, we show that the epigenetic prion switch [*SWI*^+^] establishes a specialized subpopulation with a “pioneer” phenotypic program in *Saccharomyces cerevisiae*. Cells in the pioneer state readily disperse in water, enabling them to migrate and colonize new territory. Pioneers are also more likely to find and mate with genetically diverse partners, as inhibited mating-type switching causes mother cells to shun their own daughters. In the nonprion [*swi*^*−*^] state, cells instead have a “settler” phenotype, forming protective flocs and tending to remain in their current position. Settler cells are better able to withstand harsh conditions like drought and alkaline pH. We propose that these laboratory observations reveal a strategy employed in the wild to rapidly diversify and grant distinct, useful roles to cellular subpopulations that benefit the population as a whole.

## Author summary

Microbial species do not have control over their environment. In order to survive changes (for example, in temperature, pressure, humidity, or nutrient levels), microbes must either migrate to more habitable locations or adapt to better tolerate the changing environment. Such adaptation is typically thought to come about through changes in the genetic code. Yet an alternative path also exists. In baker’s yeast, prion switching allows cells to acquire new states that may each be better adapted to different environments. Prion switching is reversible and involves no genetic change, instead relying on alternate heritable protein conformations. In this study, we show that a particular yeast prion called [*SWI*^+^] enhances the ability of cells to disperse in flowing water and also favors mating with dissimilar partners, promoting genetic diversification. Therefore, in nature, this prion could assist yeast to both migrate and adapt to new environments. Converting to this “pioneer” state comes with a trade-off: cells harboring [*SWI*^+^] are less fit in all stressful environments we tested. Thus, the prion switch serves to specialize cells either into “pioneers” that explore more physical and genetic space or into “settlers” that tend to remain where they are and are better able to survive harsh conditions.

## Introduction

Microbes face a constant struggle for resources and survival in an environment that changes by the hour. Wide temperature fluctuations, severe drought, and other environmental factors can cause mass extinction events, leaving vast regions for surviving cells to recolonize. Likewise, events such as floods or fruit falling from trees may fill new areas with nutrients, enabling expansion and growth of microbial populations. However, exploration of a new area with unknown resources carries a significant risk of death. How have microbes evolved to spread efficiently without excessive risk?

Prions are powerful evolutionary devices by which yeast cells switch between phenotypic programs, “hedging their bets” to survive in fluctuating and unpredictable environments [1–6]. Prion proteins can exist in at least two stable conformations, each having an altered function—typically a soluble, high-activity “[*prion*^*−*^] state” and an aggregated, low-activity “[*PRION*^+^] state.” A cell will generally contain one conformation for each prion protein, and these conformational states switch at rates that range from 10^−2^ to 10^−7^ cells per generation [2]. At least 10 different proteins in *S*. *cerevisiae* have the capacity to propagate in alternate prion states [6–9]. Thus, the combination of potential prion states can lead to a plethora of switchable phenotypes.

An advantage of prion-based phenotypes is that they can be totally reversed by a return to the original protein conformation. In contrast, a new phenotype gained by genetic mutation is very difficult to perfectly reverse, particularly in the case of loss-of-function mutations and deletions. Prion-based adaptation is thus ideal for flexibly changing between different phenotypic programs that are each advantageous in different situations [10]. The [*SWI*^+^] prion has previously been shown to cause slow growth in nonfermentable carbon sources [11] and a loss of invasiveness and flocculation [12] but has not previously been demonstrated to provide any advantage to yeast.

The protein that underlies the [*SWI*^+^] prion state, Swi1, is a DNA-binding protein and subunit of the SWI/SNF chromatin remodeling complex. In laboratory strains and conditions, the [*SWI*^+^] prion stochastically arises at frequency of approximately 10^−3^–10^−5^ [13] and can be lost after prolonged treatment in stressful conditions [14]. The [*SWI*^+^] prion was recently shown to regulate adhesion, invasion, pseudohyphal growth, and flocculation through a prominent effect on the expression level of flocculin genes [12]. In the [*SWI*^+^] state, *FLO1* and *FLO11*, and thus flocculation and invasion, are greatly repressed. Could this loss of multicellularity confer advantages in microbial migration?

## Results and discussion

**Fig 1:**
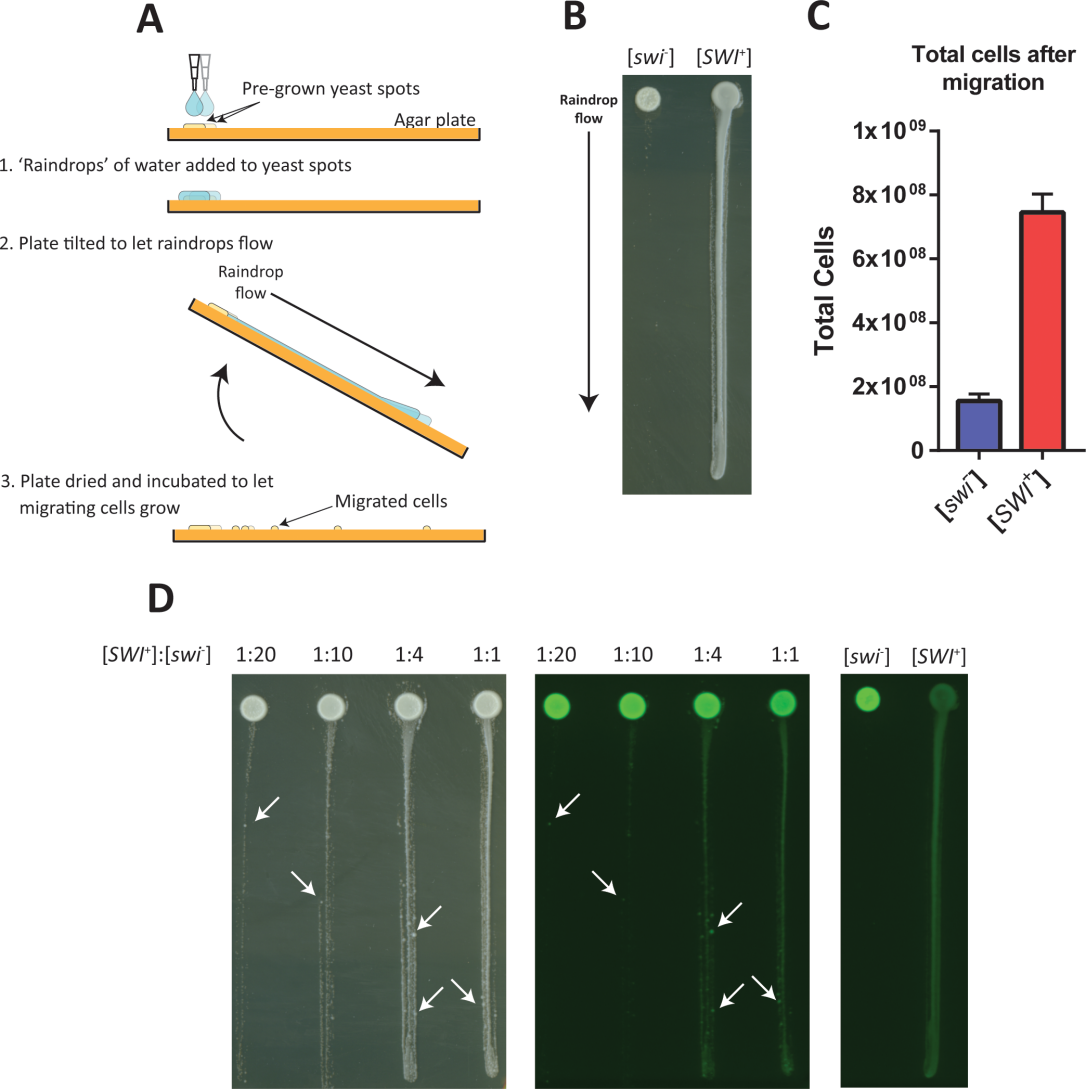
The [*SWI*^+^] prion enhances the migration of cells with the flow of water. (A) A diagram of the experimental procedures used to test the migration of cells on solid media. Pregrown spots of yeast colonies experience “rainfall” as drops of water are pipetted over them. The plates are tilted to allow the water to flow across the surface. After drying and incubating for yeast growth, colonies established by the migrated cells appear. (B) Photograph showing the migration of [*swi*^*−*^] and [*SWI*^+^] cells on an agar plate. (C) Quantification of total cell number after the migration experiment. Error bars indicate standard deviation (*n* = 3). Numerical data are available from the Dryad Digital Repository: http://dx.doi.org/10.5061/dryad.d5r16. (D) Left: Photograph showing the migration of cells on an agar plate after [*SWI*^+^] and [*swi*^*−*^] cells were mixed in the indicated proportions. Arrows indicate large colonies where small flocs of [*swi*^*−*^] cells have migrated. Center: The same image collected in a green fluorescence channel. Due to the yTRAP sensor [15] in both strains, [*swi*^*−*^] colonies are brightly fluorescent. Right: Migration experiment from panel B photographed in the green fluorescence channel for comparison. yTRAP, yeast transcriptional reporting of aggregating proteins.

One likely means of microbial migration is the flow and agitation of water, especially during rainfall. We designed experiments to test the effect of [*SWI*^+^]-dependent flocculation on migration under rain-like conditions. [*SWI*^+^] and [*swi*^*−*^] strains were spotted side by side on agar plates. After one day of growth on solid substrate, small droplets of water were added onto each spot of yeast and the plate was tipped to one side to allow the water droplet to flow down its surface (Fig 1A). A multichannel pipette was used to ensure equal “rainfall” on each spot. Plates were dried and photographed after one more day of growth (Fig 1B). The [*SWI*^+^] prion conferred a clear advantage for the spreading of yeast away from the original spot, allowing cells to colonize the entire area touched by flowing water. We expect this phenomenon would also occur in nature—testing migration on natural substrates under actual rainfall would be an interesting future experiment. In this laboratory setting, scraping each lane of migrated cells separately into solution and subsequent measurement by flow cytometry revealed that there were about four times more [*SWI*^+^] cells than [*swi*^*−*^] cells at the conclusion of this experiment (Fig 1C), demonstrating the potential growth advantage of a “pioneer” population. This migratory enhancement is specifically due to the prion’s effect mediated through the flocculin genes. In a strain lacking a transcription factor required for expression of flocculins (*flo8*^-^), the [*SWI*^+^] prion does not affect migration (S1A Fig).

Could a small population of [*SWI*^+^] cells confer a migratory benefit to their [*swi*^*−*^] neighbors? It is logical to expect that [*SWI*^+^] cells might disrupt larger flocs that would otherwise be formed by adjacent [*swi*^*−*^] partners, thereby encouraging the escape and migration of [*swi*^*−*^] cells with flowing water. We tested for such a cooperative migratory effect by seeding different ratios of [*SWI*^+^] and [*swi*^*−*^] cells on agar plates. The same simulated rainfall procedure was applied, and migrated cells were grown for one day and imaged (Fig 1D). Only when [*SWI*^+^] and [*swi*^*−*^] populations were mixed did larger colonies appeared within the migrants; these were likely formed by the migration of clusters of cells (Fig 1D, arrows). To confirm the prion state of these colonies, we utilized the yeast transcriptional reporting of aggregating proteins (yTRAP) sensor for Swi1 [15], which yields bright fluorescence in [*swi*^*−*^] cells and dim fluorescence in [*SWI*^+^] cells. The large migrated colonies from mixed populations were indeed brightly fluorescent and thus [*swi*^*−*^] (Fig 1D, left and center, arrows). In contrast, when [*SWI*^+^] and [*swi*^*−*^] were spotted separately, no bright, [*swi*^*−*^]-clustered cells migrated (Fig 1D, right). The relative amount of [*SWI*^+^] cells seeded in this experiment were higher than one may expect to arise spontaneously [13]. However, when rare, nonadherent [*SWI*^+^] cells disperse in flowing water, they will establish new populations that originate as 100% [*SWI*^+^]. Such populations, over time, may experience the full range of [*SWI*^+^]:[*swi*^*−*^] ratios tested in this experiment. We conclude that [*SWI*^+^] not only migrates more efficiently itself but also can cooperatively enhance the migration of otherwise immobile [*swi*^*−*^] neighbors.

In liquid growth media, the [*SWI*^+^] prion has a noticeable effect on the partitioning of cells into clumped flocs visible with the naked eye (dominated by [*swi*^*−*^] cells) or the suspended “supernatant” (dominated by [*SWI*^+^] cells) [12]. We quantified this effect using yTRAP—[*SWI*^+^], and [*swi*^*−*^] cells were co-inoculated in equal number and grown for 16 hours. We then used flow cytometry to measure the prion state of cells in the supernatant versus the total culture after dispersion of flocs (S1B Fig). The [*SWI*^+^] cells were generally less fit and comprised less than 40% of the total culture. However, in the supernatant fraction, [*SWI*^+^] cells comprised more than 70%. When cultures were grown in the presence of ethanol, a byproduct of yeast growth that is known to enhance flocculation [16], the fraction of [*SWI*^+^] cells in the supernatant increased to over 90%. We hypothesized that these supernatant cells would be more capable of migration, as they would overflow during rainfall. We tested the overflow of liquid cultures in a laboratory setup (S1C Fig). Indeed, [*SWI*^+^] cells had a strong advantage in escaping to colonize new ground (S1D Fig). These results establish a clear benefit to [*SWI*^+^] cells in migration in response to the common environmental stimulus of flowing or falling water. We therefore term [*SWI*^+^] cells “pioneers” and the nonmigratory [*swi*^*−*^] cells “settlers”.

Swi1’s effect on transcription is not limited to flocculins. In fact, the *SWI1* gene is so named because its mutation leads to a defect in transcription at the *HO* locus, which is required for mating-type switching. Mating-type switching occurs after a diploid cell has sporulated to produce four haploid spores. Haploid cells can mate with one another to return to the more robust diploid state, but only if the two mating partners are of opposite mating types. Spores containing a functional *HO* gene will commonly divide once or twice, after which the mother cells switch their mating type and mate with their own daughter cells, generating homozygous diploids [17]. While this does not increase genetic diversity, it does return both cells to the more robust diploid state without the risks of out-crossing (for example, the risks of acquiring transposons, 2 μ DNA, or nonadaptive prion states). Because mating-type switching occurs readily in the lab, it is a mystery as to why so many natural *S*. *cerevisiae* isolates are heterozygous and appear to have mated most recently with diverse partners (out-crossing) rather than daughter cells [18].

**Fig 2:**
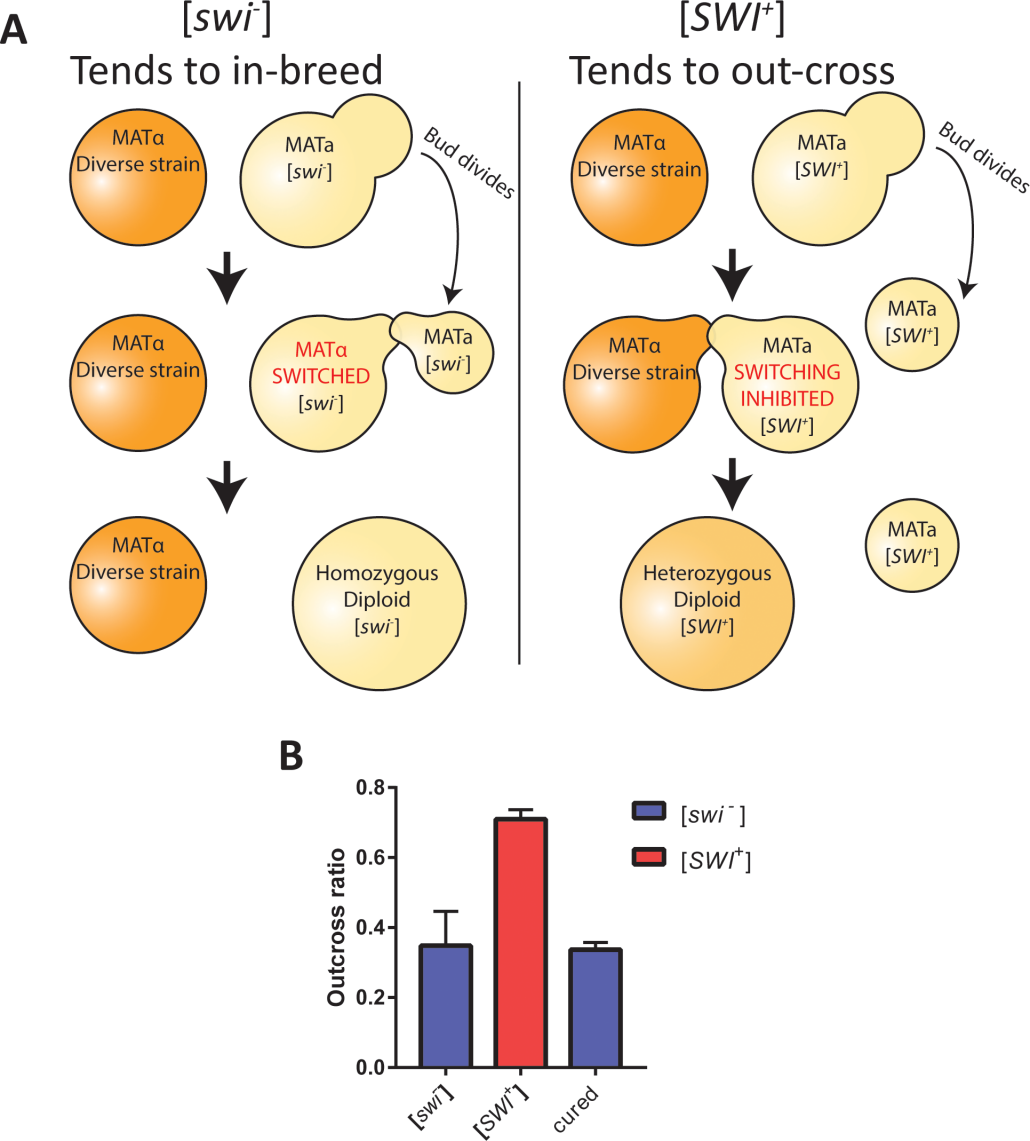
The [*SWI*^+^] prion encourages diverse mating partners. (A) A diagram showing the mating tendencies of [*SWI*^+^] and [*swi*^*−*^] haploids (budding yellow cell). Left: A [*swi*^*−*^] cell will readily switch its mating type after dividing and mate with its own daughter. The resulting diploid cannot out-cross with diverse mating partners (orange cell). Right: In the [*SWI*^+^] pioneering state, mating-type switching is inhibited, and thus mother cells cannot mate with their own daughters. This increases the likelihood that they will mate with genetically diverse partners. Note that after normal mating-type switching, an additional generation may occur before mating, after which the four haploids involved can mate in pairs (not depicted). (B) Relative out-crossing efficiencies of [*swi*^*−*^] cells (blue) and [*SWI*^+^] cells (red). Elimination of the prion from the [*SWI*^+^] strain (“cured”) returns its out-cross ratio to the low state. The ratio was calculated by normalizing to the mating efficiency of *ho*^*−*^ control cells (S2A Fig). Error bars indicate standard deviation (*n* = 3). Numerical data and the flow cytometry gating strategy are available from the Dryad Digital Repository: http://dx.doi.org/10.5061/dryad.d5r16.

We hypothesized that the [*SWI*^+^] prion, like *SWI1* mutants, may confer a defect in *HO* expression and thereby increase out-crossing frequency. To test this, we measured the out-crossing efficiency of [*SWI*^+^] and [*swi*^*−*^] *HO*^+^ spores. Our measurement was normalized to identical *ho*^*−*^ control spores to specifically determine the prion effects mediated through *HO* (experimental procedure: S2 Fig). As anticipated, the [*SWI*^+^] prion increased the out-crossing efficiency of *HO*^+^ spores (Fig 2). Elimination of the prion by passaging on guanidine [11] returned the out-crossing frequency to that of the original [*swi*^*−*^] strain, confirming that the effect was indeed mediated through the prion state. Thus, the [*SWI*^+^] prion acts as a deterrent for inbreeding and encourages the diversification of genomes by out-crossing. We propose that a combined effect of enhanced migration, which would increase the chance of encountering diverse strains, with enhanced out-crossing would synergistically favor the generation of diverse hybrid diploids by pioneer cells.

**Fig 3:**
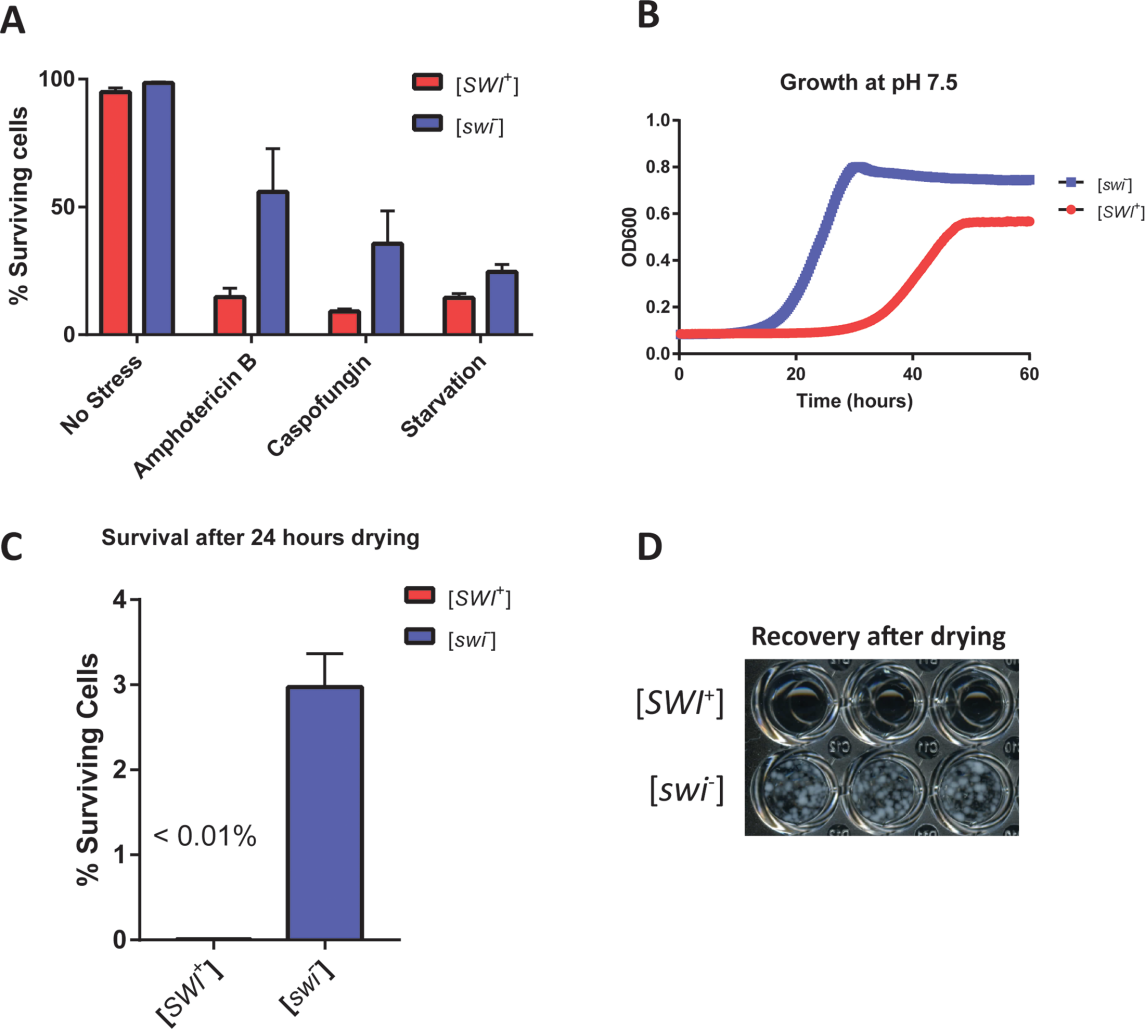
[*SWI*^+^] cells have lower fitness and do not survive dry conditions. (A) The survival of [*SWI*^+^] (red) and [*swi*^*−*^] (blue) cells from overnight cultures (no stress), 6-day–starved cultures (starvation), and antifungal-treated overnight cultures (amphotericin B and caspofungin) were measured using a viability stain and flow cytometry. Error bars indicate standard deviation (*n* = 3). All numerical data in this figure and the flow cytometry gating strategy are available from the Dryad Digital Repository: http://dx.doi.org/10.5061/dryad.d5r16. (B) Growth comparison of [*swi*^*−*^] cells and [*SWI*^+^] cells in media buffered at pH 7.5. Cell density was measured by absorbance every 15 minutes. (C) Survival of [*SWI*^+^] and [*swi*^*−*^] cultures after removal of liquid media followed by drying under blowing air for 24 hours. The survival of [*SWI*^+^] was below the detection limit using viability stain and flow cytometry (1 in 10,000 cells). Error bars indicate standard deviation (*n* = 3). (D) Recovery of dried [*SWI*^+^] and [*swi*^*−*^] strains in liquid culture in microtiter plates. After rehydration of dried cells into the original volume of the liquid culture, a 25-fold dilution into fresh media was made in microtiter wells. Growth after four days is depicted.

The [*SWI*^+^] prion is acutely sensitive to perturbations in the Hsp70 chaperone system. Proteotoxic stress such as elevated temperature and the modulation of certain chaperones has been shown to entirely eliminate [*SWI*^+^], but not the prions [*PSI*^+^] or [*RNQ*^+^] [14]. How might cells benefit from eliminating [*SWI*^+^] in response to stress? To test this, we compared the survival and growth of [*SWI*^+^] and [*swi*^*−*^] strains across a number of conditions (Fig 3). In every condition tested, the “settler” [*swi*^*−*^] state was advantageous. After six days of starvation or one day of antifungal treatment (caspofungin or amphotericin B), the flocculated [*swi*^*−*^] cells showed increased survival (Fig 3A). The growth defect of [*SWI*^+^] cells was particularly dramatic when media was buffered to pH 7.5 (Fig 3B) but was also present when cells were grown at a favorable pH supplemented with glucose or raffinose (S3 Fig). [*swi*^*−*^] cultures were also uniquely able to withstand dry conditions—after 24 hours under a draft of sterile air, about 3% of [*swi*^*−*^] cells survived, while the survival of [*SWI*^+^] cells was below the detection limit (Fig 3C). Upon rehydration and subsequent incubation, [*swi*^*−*^] cells recovered and grew in microtiter wells, while [*SWI*^+^] cells did not (Fig 3D). This demonstrates the advantage of the settler phenotype and provides an evolutionary rationale for switching to the [*swi*^*−*^] settler state under stress.

Our results suggest an environmental factor that is likely to play a major role in the relative advantage of the [*SWI*^+^] or [*swi*^*−*^] phenotypic states: moisture. Rainfall, dew, or other sources of fluid motion could favor [*SWI*^+^] pioneers that readily disperse in water, colonizing new territory and mating with diverse partners. Dry conditions, on the other hand, may eliminate these advantages and instead favor [*swi*^*−*^] settlers that are resistant to desiccation.

Notably, the human homolog of Swi1, ARID1A, is commonly lost in multiple types of cancer [19]. Loss of ARID1A confers increased cellular migration and metastases and is associated with poor prognosis [20, 21]. This effect is mediated by a resulting loss of E-cadherin [21], a cell surface protein that would otherwise promote cell–cell adhesion in epithelia [22, 23]. In a reminiscent manner, [*SWI*^+^] mimics a Swi1 loss-of-function in yeast, leading to reduced expression of flocculins and a loss of adhesion to surrounding cells and substrates [12]. This results in the increased ability of [*SWI*^+^] cells to migrate. Although yeast flocculins, regulated by Swi1, and human E-cadherin, regulated by ARID1A, are not from the same protein family, both form calcium-sensitive cell–cell adhesions. Our discovery that [*SWI*^+^] regulates yeast migratory states may point to a conserved role of Swi1 family members in controlling cell adhesion and thus migration.

The process of cell migration is of great importance to single-celled organisms, as nutrient availability can vary widely from place to place. The bacterium *Caulobacter crescentus* deterministically encodes a switch between specialized motile and stationary phenotypic programs within its cell cycle [24]. This presumably assists the organism to colonize new areas without the risk of abandoning its present location. A few other specialized states in microbes are known to switch in a stochastic manner, for example, bacterial persister cells that survive antibiotic treatment and white/opaque switching in *Candida albicans* that regulates aspects of host interactions and mating [25, 26].

Prions are thought to act as bet-hedging elements in *S*. *cerevisiae* [1, 3, 4, 6, 9]. That is, they confer environment-specific growth advantages so that a diverse population with many prion states—and thus many hedged bets—is more likely to have subsets of cells that are already adapted to unpredictable future environments. The [*SWI*^+^] prion has not previously been shown to confer a growth advantage in any environment, so its function as a potential bet-hedging element was mysterious. We have demonstrated that [*SWI*^+^] can indeed benefit the growth of the total population when the opportunity to migrate arises (Fig 1C). We propose that, in addition to simple bet-hedging of growth rates, prions can generate specialized subpopulations of cells with the ability to take advantage of unique ecological niches. Based on the results presented here, we hypothesize that wild [*SWI*^+^] cells would have an advantage over isogenic [*swi*^*−*^] neighbors in colonizing new territory and sampling diverse genetic space though mating, thus acting as specialized “pioneers” on both fronts. In dry or otherwise stressful natural environments, we predict that [*swi*^*−*^] cells will have a survival advantage and that [*SWI*^+^] cells will switch to the [*swi*^*−*^] state at increased rates. The [*swi*^*−*^] “settler” phenotype could also be advantageous in allowing cells to adhere to and remain in favorable locations, even during rainfall that would wash pioneer cells away. The first bacterial prion was recently discovered [27], proving that the ability to switch phenotypes based on prion propagation is not limited to yeast. Prion-based specialization strategies could be widely used in single-celled organisms to switch between risky yet potentially beneficial phenotypic programs.

## Materials and methods

### Cloning and vector construction

The yTRAP sensor plasmid pGAN202 [15] was used to detect the [*SWI*^+^] prion. This plasmid integrates into the *HO* locus, disrupting the endogenous site and adding a fluorescent detection cassette for the [*SWI*^+^] prion. It produces bright green fluorescence in the [*swi*^*−*^] state and dim fluorescence in the [*SWI*^+^] state.

The multicopy, episomal plasmid pGAN160 (Newby et. al., in review) was used to induce the [*SWI*^+^] prion in cells by overexpression of Swi1 from the *GAL1* promoter in galactose.

The *FLO8*-correction plasmid pGAN300 was made using pAG305GAL [28]. pAG305GAL was cut with the restriction enzymes XbaI and XhoI, followed by ligation of the wild-type *FLO8* coding sequence. The restriction enzyme BstAPI was used to cut the plasmid between the start codon of *FLO8* and the site of its mutation in laboratory strains to favor correction of the endogenous copy under its native promoter.

### Yeast strains and growth media

Yeast strains used in this study were derivatives of BY4741 [29] (*his3d1*, *leu2Δ*, *met15Δ*, *ura3Δ*, *ho*^*−*^). BY4741 *trp1*:*KanMX* from the Invitrogen deletion library (Cat. no. 95401.H2) and SK1 [30] (*HO*^*+*^ *a/α)* were used to determine out-crossing efficiency. To restore flocculation in BY4741, the *FLO8* gene was corrected using pGAN300. For a complete list of strains, please see S1 Table.

Growth media was YPD or complete standard synthetic media (CSM) or media lacking certain amino acids with either 2% glucose, 2% galactose, or 2% raffinose supplemented as the carbon source. [*SWI*^+^] was cured by streaking on YPD agar plates supplemented with 5 mM guanidine hydrochloride. Yeast transformations were conducted using a lithium acetate competent cell protocol as previously described [31]. To calculate the OD600 of flocculating cells or measure them by flow cytometry, flocs were fully dispersed by the addition of 25 mM EDTA followed by vigorous vortexing or pipetting.

To induce the [*SWI*^+^] prion, the Swi1 overexpression plasmid pGAN160 was transformed into a strain containing the yTRAP sensor (pGAN202). The Swi1 protein was transiently overexpressed by growth for 16 hours in galactose followed by plating on glucose. Potential [*SWI*^+^] colonies had heritable, low fluorescence. The presence of the prion was confirmed by streaking on guanidine media—if the strain was [*SWI*^+^], this treatment returned cells to the bright, [*swi*^*−*^] state.

### Migration from liquid culture

To test for the ability of cells to emerge from an overflowing liquid pool, 2-tiered holes were produced in agar YPD medium. Overnight, 50-μL cultures of CSM + 2% glucose + 5% ethanol (strains yGAN102 and yGAN103) were pipetted into the bottom hole and allowed to settle for one hour. A multichannel pipette was used to transfer 800 μL of sterile water into each hole simultaneously so that the bottom tier overflowed onto the top. Then, the water was aspirated away and the plates were dried under flowing air in a hood for one hour, then transferred to 30 ^o^C to allow colonies to grow. Migrated colonies were photographed on a Bio-Rad ChemiDoc.

In order to make the 2-tiered holes, a custom mold was ordered from the MIT machine shop made from PDMS. Six pins were inserted into the mold, each of which formed a 2-tiered hole. The bottom-tier hole had a radius of 0.15 in and a height of 0.47 in. The top hole had a radius of 0.9 in and a height of 0.47 in. Each pin was placed 1.4 in apart. The sides of each tier were slanted at 6 ^o^ to ease removal from solidified agar.

### Supernatant/Total culture quantification

Quadruplicate, 4 mL YPD or YPD + 5% ethanol cultures were inoculated with 50% [*SWI*^+^] (yGAN103) and 50% [*swi*^*−*^] (yGAN102) to a final OD600 of 0.02. Cultures were grown for 16 hours at 30 ^o^C with shaking. Tubes were let sit for 15 minutes on the bench without agitation. Supernatant samples were collected from the top of the culture (approximately 0.1 OD600 units), and cultures were then fully resuspended with EDTA to disperse flocs. Additional samples were collected to represent the total culture. All samples were centrifuged, resuspended in 500 μL fresh YPD, and incubated four hours at 30 ^o^C in deep well 96-well plates to reach log phase before measurement. EDTA was added to disperse flocs, and samples were measured by flow cytometry using the MACSquant VYB (Miltenyi Biotec). The percentage of [*SWI*^+^] cells was calculated by gating in the GFP/SSC channels.

### Migration on solid agar plates

Overnight cultures of yGAN102 and yGAN103 (OD600 ~ 8) were spotted on rectangular YPD plates. After 16 hours of growth at 30 ^o^C, 14 μL of sterile water was pipetted on the spots using a multichannel pipette to ensure even flow. Plates were immediately tipped to the side until vertical, allowing the drops of water to flow down the surface of the plate. Plates were incubated 16 hours more at 30 ^o^C, then photographed. The Bio-Rad ChemiDoc was used to collect images in the green fluorescent channel. An Epson document scanner was used to take normal photographs.

To calculate the total number of cells after migration, the original yeast spot and all yeast that grew on its trail of migration were scraped into 500-μL CSM. This was diluted 100-fold and measured by flow cytometry as above. The number of cells per μL was used to calculate the total number of cells that were present on the plate.

For mixtures of [*SWI*^+^] and [*swi*^*−*^] cultures, EDTA was added to all cultures to disperse flocs (for consistency, nonflocculating [*SWI*^+^] cells were also treated with EDTA). ODs were normalized, and cultures were mixed in the given ratios then centrifuged and resuspended in media lacking EDTA. These were spotted onto agar plates as above.

### Out-cross ratio measurement

The procedure to calculate the out-cross ratio is summarized in S2 Fig. The procedure is relatively involved because *HO*^*+*^ haploids cannot be propagated stably in the laboratory. The homozygous, *HO*^*+*^ diploid SK1 strain was sporulated by overnight incubation in 2% potassium acetate. SK1 spores were mated to *MATa flo8-*[*SWI*^+^] and -[*swi*^*−*^] strains (yGAN100 and yGAN101) by co-inoculation into YPD and overnight growth. Hybrid diploids were selected by growth on media lacking histidine, supplemented with 100 μg/mL nourseothricin. The [*SWI*^+^] diploid was cured by passaging on guanidine to generate an additional control for the effect of the prion. This generated yeast strains yGAN104-106 (S1 Table).

These hybrid diploids contained one functional copy of *HO* (from SK1) and one mutant copy (from the [*SWI*^+^] sensor strain). Importantly for a later selection step, these diploids were homozygous *TRP1*^*+*^. Due to the *HO*-integrated pGAN202, the mutant *ho−* allele is marked with the expression of a green fluorescent protein. Upon sporulation, the green *ho−* spores will be preferentially able to mate with an exogenous strain. *HO*^*+*^ nonfluorescent spores are instead able to switch mating types and mate with their daughters or other near neighbors. To test whether the [*SWI*^+^] prion affects this tendency, we sporulated the diploids for seven days in 2% potassium acetate, followed by vigorous vortexing. We then mixed triplicate mating tubes of YPD containing these spores and BY4741 *trp1*:*KanMX* at an OD600 of 0.01 each and final volume of 4 mL. Mating tubes were incubated at 30°C for 16 hours.

Diploids formed between the exogenous strain (BY4741 *trp1*:*KanMX*) and any spores were selected by 20-fold dilution into synthetic media lacking tryptophan and supplemented with 100 μg/mL geneticin (Sigma Aldrich A1720). After 24 hours of growth, selection was continued with a further 100-fold dilution into the same selective media. After another 24 hours of growth, cultures were diluted 100-fold in the same media and grown six hours to log phase before flow cytometry measurement as above. PI stain was used to exclude dead cells. The out-cross ratio was calculated as the number of nonfluorescent diploids (formed from *HO+* spores) divided by the number of fluorescent diploids (formed from *ho−* spores). This allowed us to normalize specifically to the effect of the [*SWI*^+^] prion mediated through the *HO* locus.

### Survival measurement

Small colonies of *FLO8+* [*swi*^*−*^] or [*SWI*^+^] strains (yGAN102 and yGAN103) were picked into triplicate cultures of synthetic complete media. 150 ng/mL caspofungin (Abcam ab145180) or 5 μg/mL amphotericin B (Santa Cruz Biotechnology sc-202462) were used for antifungal treatment—these two drugs comprise the two major classes of naturally occurring fungicidal compounds. After overnight growth (or one week of incubation in the case of starved cultures), cultures were centrifuged and resuspended in fresh CSM. 25 mM EDTA was added to disrupt flocs, and 10 μg/mL propidium iodide (Sigma Aldrich Cat. no. P4864) was added to stain dead cells. Cultures were measured by flow cytometry and dead/live cells were gated in the red fluorescence channel.

To test survival in dry conditions, overnight 4-mL cultures of the same strains were pelleted by centrifugation for four minutes at 2,000 rpm, following which the supernatant was discarded. The plastic culture tubes containing [*swi*^*−*^] or [*SWI*^+^] cell pellets were left without caps in a sterile hood, with air blowing over them for 24 hours. After this desiccation period, 4 mL of fresh media was added to rehydrate cells. Then, the same survival measurement was conducted as above, with the addition of EDTA and propidium iodide followed by flow cytometry. To test recovery from drying out, these cultures were separately diluted 25-fold into fresh media and left in microtiter plates for four days to recover before imaging with a standard camera.

### Growth curve determination

Growth curves were collected using a ThermoFisher MultiSkan GO at 30 ^o^C. Cells were diluted to OD600 of 0.01 in the indicated media. Absorbance at 600 nm was collected every 15 minutes after 15 seconds of shaking. For pH 7.5 media, pH was buffered with 100 mM sodium phosphate. Strains used for growth curve determination were *flo8*^*−*^ (yGAN100 and yGAN101) because flocculation interferes with absorbance measurements.

### Data and flow cytometry gating strategy availability

Numerical data used to generate graphs in this article, along with a depiction of the gating strategies used to analyze flow cytometry data, are available from the Dryad Digital Repository: http://dx.doi.org/10.5061/dryad.d5r16 [32].

## Supporting information

S1 Fig *FLO8*-dependent migratory benefits of [*SWI*^+^] cells. (A) Comparison of migration of [*SWI*^+^] and [*swi*^*−*^] cells lacking a functional *FLO8* gene, which is required for the expression of Flo1 and Flo11 [12]. (B) Fraction of the total liquid culture (notched bars) or supernatant only (plain bars) that is [*SWI*^+^] after 16 hours of growth in YPD or YPD + 5% ethanol initiated at equal inoculum of [*SWI*^+^] and [*swi*^*−*^] cells. Measurements were made using flow cytometry on the yTRAP sensor that reports on prion status by fluorescence [15]. Flocs were disrupted chemically using EDTA to solubilize cells for measurement. Error bars indicate standard deviation from quadruplicate cultures. Numerical data and the flow cytometry gating strategy is available from the Dryad Digital Repository: http://dx.doi.org/10.5061/dryad.d5r16 (C) A diagram of experimental procedures to test the ability of cells to migrate in liquid culture. Yeast cultures are inoculated at the bottom of a two-tiered agar well. Water is added until it overflows onto the upper tier, followed by aspiration of the water. After incubation to allow cell growth, colonies established by migrated cells appear on the upper tier. (D) Photograph comparing the migration of [*SWI*^+^] and [*swi*^*−*^] cells in liquid media. [*SWI*^+^] cells migrate so efficiently that they form a lawn of colonies on the upper tier. YPD, yeast extract, peptone, and dextrose; yTRAP, yeast transcriptional reporting of aggregating proteins.
(TIF)

S2 Fig Experimental procedure for out-cross ratio measurement. [*SWI*^+^] and [*swi*^*−*^] diploid strains were sporulated. Each had the genotype *HO*^*+*^*/ho*^*−*^, where the *ho* locus was marked with a cassette expressing the green fluorescent protein NeonGreen. A large pool of these spores was diluted into mixed culture with a *ho*^*−*^, selectable, haploid tester strain. After allowing ample time for mating to occur, we selected for hybrids formed by mating events between the spores and the tester strain. We then determined the ratio of nonfluorescent *HO*^+^ spores that out-crossed with the tester strain to fluorescent *ho*^*−*^ spores that out-crossed.
(TIF)

S3 Fig [*SWI*^+^] pioneers are less fit than [*swi*^*−*^] settler cells. (A) Growth comparison of [*swi*^*−*^] cells (blue) and [*SWI*^+^] cells (red) in standard growth media supplemented with glucose, or B) raffinose. Cell density was measured by absorbance at 600 nm every 15 minutes. Numerical data is available from the Dryad Digital Repository: http://dx.doi.org/10.5061/dryad.d5r16.
(TIF)

S1 Table Yeast strains used in this study. Each yeast strain used in the study is listed along with its Swi1 prion status, transformed plasmids, and genotype.
(XLSX)

## Abbreviations

CSM: complete standard synthetic media
YPD: yeast extract, peptone, and dextrose - a rich media for yeast growth
yTRAP: yeast transcriptional reporting of aggregating proteins
