# Supplementary figures and images for: Pioneer cells established by the [SWI+] prion can promote dispersal and out-crossing in yeast
Source: PLoS Biol. 2017 Nov 14;15(11):e2003476. doi: 10.1371/journal.pbio.2003476 (PMC5685480; doi:10.1371/journal.pbio.2003476)

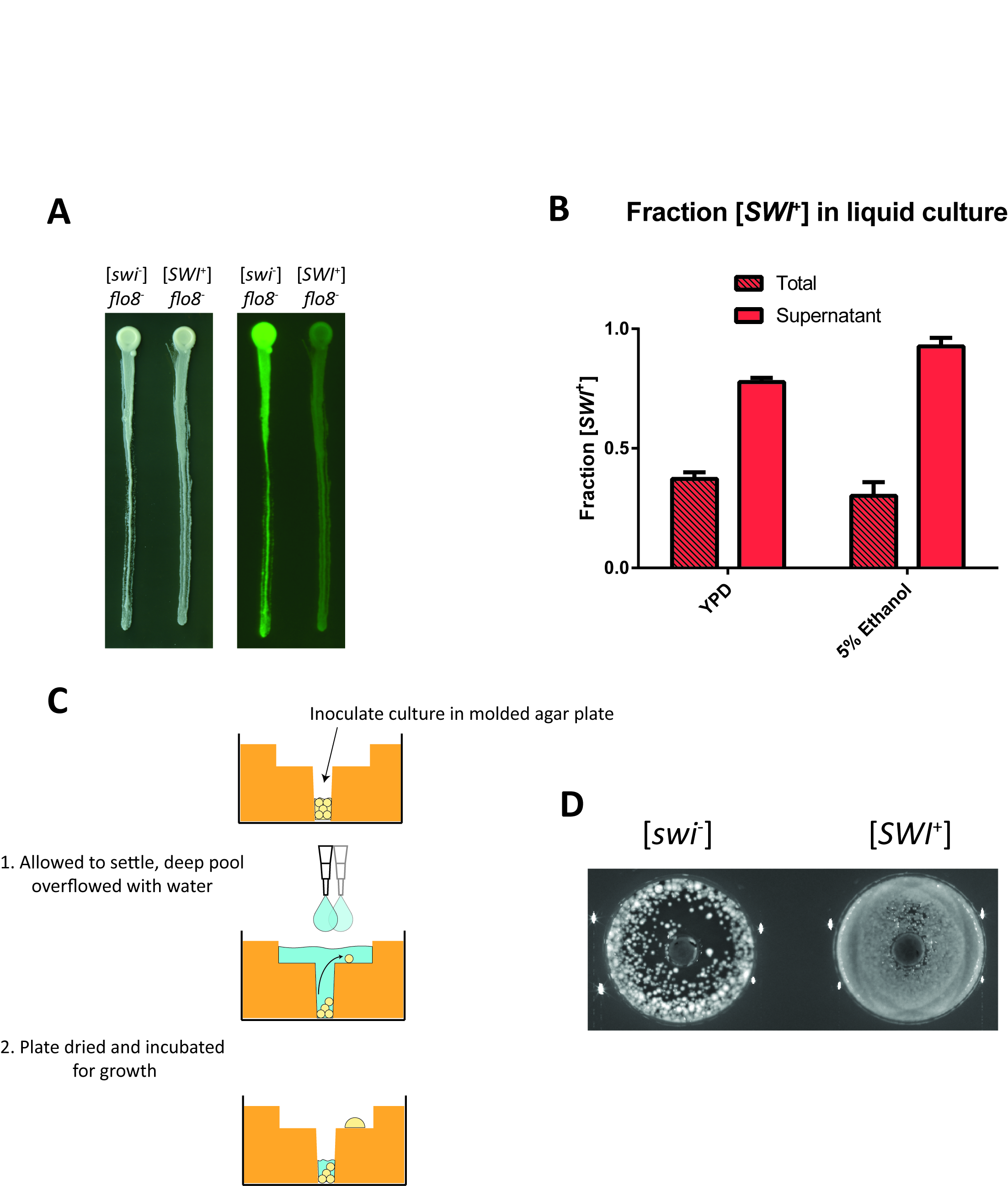

Supplement: S1 Fig — (A) Comparison of migration of [SWI+] and [swi−] cells lacking a functional FLO8 gene, which is required for the expression of Flo1 and Flo11 [12]. (B) Fraction of the total liquid culture (notched bars) or supernatant only (plain bars) that is [SWI+] after 16 hours of growth in YPD or YPD + 5% ethanol initiated at equal inoculum of [SWI+] and [swi−] cells. Measurements were made using flow cytometry on the yTRAP sensor that reports on prion status by fluorescence [15]. Flocs were disrupted chemically using EDTA to solubilize cells for measurement. Error bars indicate standard deviation from quadruplicate cultures. Numerical data and the flow cytometry gating strategy is available from the Dryad Digital Repository: http://dx.doi.org/10.5061/dryad.d5r16 (C) A diagram of experimental procedures to test the ability of cells to migrate in liquid culture. Yeast cultures are inoculated at the bottom of a two-tiered agar well. Water is added until it overflows onto the upper tier, followed by aspiration of the water. After incubation to allow cell growth, colonies established by migrated cells appear on the upper tier. (D) Photograph comparing the migration of [SWI+] and [swi−] cells in liquid media. [SWI+] cells migrate so efficiently that they form a lawn of colonies on the upper tier. YPD, yeast extract, peptone, and dextrose; yTRAP, yeast transcriptional reporting of aggregating proteins. (TIF) [file pbio.2003476.s001.tif]

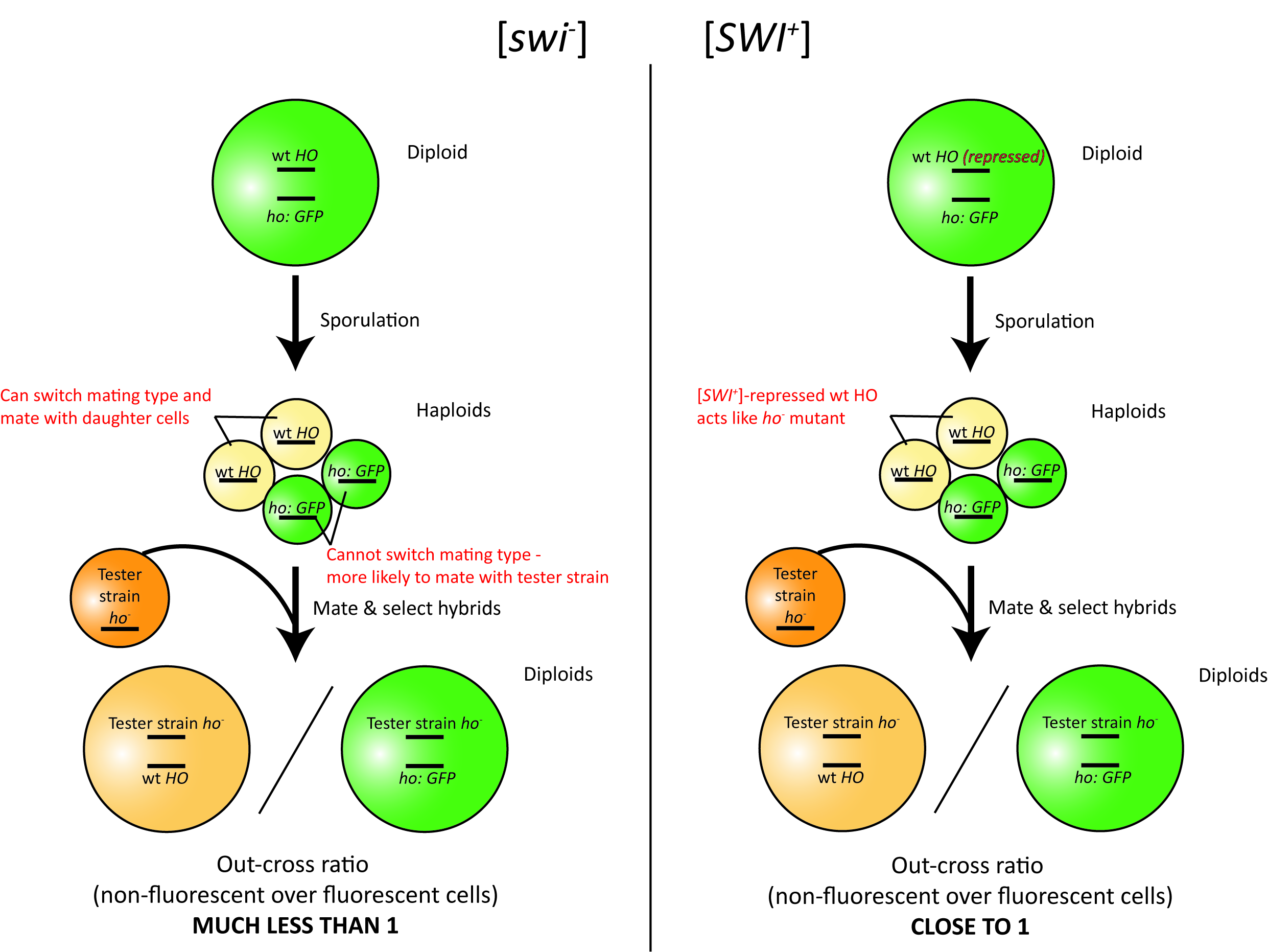

Supplement: S2 Fig — [SWI+] and [swi−] diploid strains were sporulated. Each had the genotype HO+/ho−, where the ho locus was marked with a cassette expressing the green fluorescent protein NeonGreen. A large pool of these spores was diluted into mixed culture with a ho−, selectable, haploid tester strain. After allowing ample time for mating to occur, we selected for hybrids formed by mating events between the spores and the tester strain. We then determined the ratio of nonfluorescent HO+ spores that out-crossed with the tester strain to fluorescent ho− spores that out-crossed. (TIF) [file pbio.2003476.s002.tif]

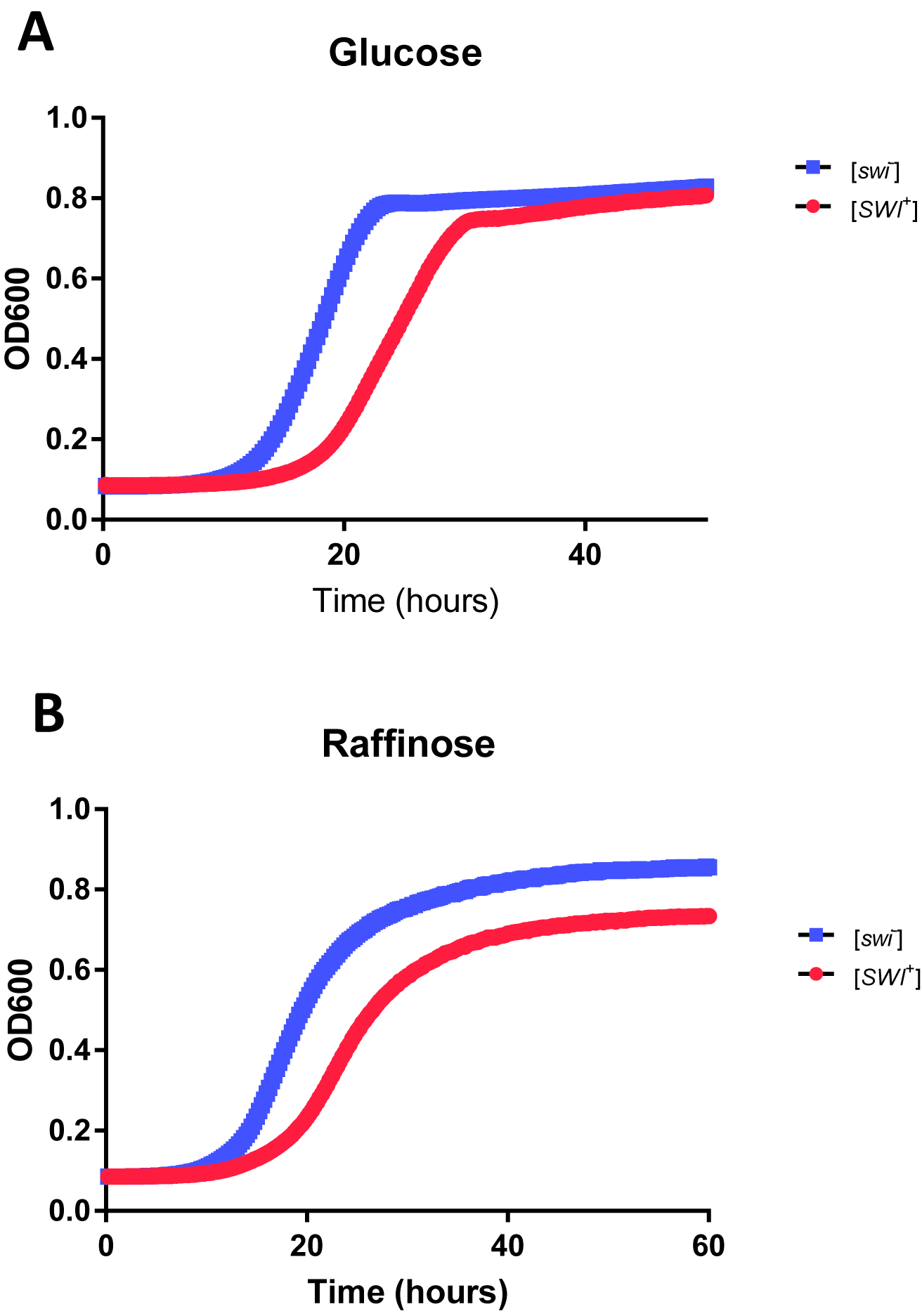

Supplement: S3 Fig — (A) Growth comparison of [swi−] cells (blue) and [SWI+] cells (red) in standard growth media supplemented with glucose, or B) raffinose. Cell density was measured by absorbance at 600 nm every 15 minutes. Numerical data is available from the Dryad Digital Repository: http://dx.doi.org/10.5061/dryad.d5r16. (TIF) [file pbio.2003476.s003.tif]
